# COVID-19 and Hospital Palliative Care – A service evaluation exploring the symptoms and outcomes of 186 patients and the impact of the pandemic on specialist Hospital Palliative Care

**DOI:** 10.1177/0269216320949786

**Published:** 2020-08-14

**Authors:** Lucy Hetherington, Bridget Johnston, Grigorios Kotronoulas, Fiona Finlay, Paul Keeley, Alistair McKeown

**Affiliations:** 1 Hospital Palliative Care Team, Queen Elizabeth University Hospital, Glasgow, NHS Greater Glasgow and Clyde, Glasgow, UK; 2 Prince and Princess of Wales Hospice, Glasgow, UK; 3 School of Medicine, Dentistry and Nursing, University of Glasgow, Glasgow, UK; 4 NHS Greater Glasgow and Clyde, Glasgow, UK; 5 Hospital Palliative Care Team, Glasgow Royal Infirmary, Glasgow NHS Greater Glasgow and Clyde, Glasgow, UK

**Keywords:** Palliative care, pandemics, terminal care, COVID-19, symptom assessment, inpatients

## Abstract

**Background:**

Patients hospitalised with COVID-19 have increased morbidity and mortality, which requires extensive involvement of specialist Hospital Palliative Care Teams. Evaluating the response to the surge in demand for effective symptom management can enhance provision of Palliative Care in this patient population.

**Aim:**

To characterise the symptom profile, symptom management requirements and outcomes of hospitalised COVID-19 positive patients referred for Palliative Care, and to contextualise Palliative Care demands from COVID-19 against a ‘typical’ caseload from 2019.

**Design:**

Service evaluation based on a retrospective cohort review of patient records.

**Setting/participants:**

One large health board in Scotland. Demographic data, patient symptoms, drugs/doses for symptom control, and patient outcomes were captured for all COVID-19 positive patients referred to Hospital Palliative Care Teams between 30th March and 26th April 2020.

**Results:**

Our COVID-19 cohort included 186 patients (46% of all referrals). Dyspnoea and agitation were the most prevalent symptoms (median 2 symptoms per patient). 75% of patients were prescribed continuous subcutaneous infusion for symptom control, which was effective in 78.6% of patients. Compared to a ‘typical’ caseload, the COVID-19 cohort were on caseload for less time (median 2 vs 5 days; *p* < 0.001) and had a higher death rate (80.6% vs 30.3%; *p* < 0.001). The COVID-19 cohort replaced ‘typical’ caseload; overall numbers of referrals were not increased.

**Conclusions:**

Hospitalised COVID-19 positive patients referred for Palliative Care may have a short prognosis, differ from ‘typical’ caseload, and predominantly suffer from dyspnoea and agitation. Such symptoms can be effectively controlled with standard doses of opioids and benzodiazepines.

**What is already known?**

- COVID-19 is a highly infectious disease with significant mortality in hospitalised patients globally, presenting new challenges for palliative care services.
- There is limited information available on prevalence of symptoms, efficacy of symptom control measures, and disease trajectory for hospitalised patients with COVID-19.

**What this paper adds?**

- Patients referred to Hospital Palliative Care Teams with COVID-19 frequently suffer with dyspnoea and agitation towards end of life and have a short dying phase.
- Evidence that symptoms can be controlled effectively in most cases with standard doses of opioids and benzodiazepines.
- Comparison data with ‘typical’ patients referred to Hospital Palliative Care Teams to inform adaptation of services to meet the needs of patients with COVID-19.

**Implications for practice, theory or policy**

- Standard doses of opioid and benzodiazepine should be used for symptom control in patients dying with COVID as per existing guidance.
- Due to a short dying phase early assessment and initiation of symptom control measures is key.
- Further research is suggested to explore the reasons for reduction in the ‘typical’ Palliative Care caseload.

## Introduction

The rapid spread of Coronavirus (COVID-19) has presented a clinical and logistical challenge to healthcare across the world. This has led to reorientation of clinical services to manage large numbers of acutely ill patients in hospital. Whilst overall mortality from COVID-19 is low, at 3% of confirmed cases, patients hospitalised with the infection have a mortality rate of 26%.^1,2^ Anecdotal reports suggest high symptom burden requiring high doses of symptom relieving medication. It was recognised that Palliative Care services would be required to adapt and form a key role in the response to COVID-19.^3^

Pre-existing literature on Palliative Care in pandemics is limited to the provision of services, rather than symptom burden and management.^4,5^ In the aftermath of the initial outbreak in Wuhan, published reports focussed on the presenting symptoms of COVID-19 rather than symptoms associated with end stage disease requiring palliation.^6^ Only recently has evidence emerged to help characterise symptom burden and outcomes in end stage COVID-19, but with small patient numbers and focussed geographical locations.^7–9^

Our primary aim was to further characterise the symptom profile, outcomes and symptom management requirements of hospitalised patients with COVID-19 referred to Hospital Palliative Care. Our secondary aim was to contextualise Palliative Care demands from COVID-19 against a ‘typical’ Palliative Care caseload pre-pandemic. This should aid understanding of the impact of COVID-19 on Hospital Palliative Care services, providing valuable information to assist in the management of further waves of the disease and pandemic planning in general.

## Methods

### Design and setting

This was a service evaluation of in-patients referred to Hospital Palliative Care in a large Scottish health board. NHS Greater Glasgow and Clyde comprises five Hospital Palliative Care Teams and four acute receiving hospitals, serving a patient population of 1.2 million in one of the most densely populated and socio-economically diverse areas in Scotland.

### Data collection and analysis

Data were collected retrospectively on all in-patients referred to Hospital Palliative Care services during a 4-week period (30th March and 26th April 2020), which included the peak for COVID-19 hospital admissions and deaths in the UK. Only patients with swab positive COVID-19 by RT-PCR nasopharyngeal swab were included.

Data were extracted from medical notes, nursing notes, drug charts electronic records and referral systems by ward-based clinicians. Variables included: demographics; comorbidities (as assessed by Charlson Comorbidity Index (CCI)); Estimated glomerular filtration rate (eGFR) at presentation; referral source; referral time frame; frequency of Hospital Palliative Care Team contact; symptoms and patient outcome.

The use of drug delivery by continuous subcutaneous infusion (CSCI) for symptom control and the drugs and final doses used were recorded. Conversion of opioid dose to subcutaneous morphine equivalence was based on a conversion of 15:1 for alfentanil and 2:1 for subcutaneous oxycodone. Symptoms and clinical impression of efficacy was sought from case note documentation in contemporaneous notes made by specialist Palliative Care clinicians throughout admission.

Comparative data from the same seasonal period pre-pandemic (1st April–28th April 2019) were extracted from service databases to explore similarities and differences in patient demographics, time on Hospital Palliative Care caseload and clinical outcomes with the COVID-19 cohort.

Univariate analysis was conducted on all data. Frequency counts were generated for nominal and ordinal variables (n, %). For interval-ratio variables, descriptive statistics were presented as median, range and interquartile range (IQR). Mann–Whitney *U* and Pearson chi-square tests were used to test for between-cohort differences in interval-ratio variables and binary/nominal variables, respectively, at the 0.05 level of significance. IBM SPSS (IBM Inc. Chicago, IL) aided the statistical analysis.

This study was registered as a service evaluation project and received clinical governance approval by NHS Greater Glasgow and Clyde and for the hospitals involved REF GGC280420.

## Results

186 patients with COVID-19 were referred to Hospital Palliative Care, accounting for 43.6% of all referrals (*n* = 427) in that time period. Despite the large number of patients with COVID-19, the total number of referrals was equivalent to the same 4-week period in 2019 (*n* = 437).

There was a slight predominance of male patients in the COVID-19 cohort (Table 1). The median age at referral was 76 [IQR 71,84]. CCI was 6 [IQR 4,7], corresponding to an estimated 10-year survival of 2%. The three most common comorbidities were hypertension (31.2%), diabetes mellitus (28%), and chronic obstructive pulmonary disease (26.9%). Presentation eGFR was >60 in 100 patients. Twenty-eight patients had an eGFR of <30.

**Table 1.** Demographics, palliative care involvement and outcomes in patients with COVID-19 referred to HPCT.

Total number of patients with COVID-19	186
Age, years; median [IQR]	76 [71,84]
Sex, Male: female (% male)	98: 88 (52.7% male)
Charlson Comorbidity Index; median [IQR]	6 [4,7]
Comorbidities; *n* (%):	
Hypertension	58 (31.2%)
Diabetes mellitus	52 (28%)
Chronic obstructive pulmonary disease	50 (26.9%)
Ischaemic heart disease	45 (24.2%)
Dementia	41 (22%)
Chronic kidney disease	34 (18.3%)
Cerebrovascular disease	29 (15.6%)
Solid tumour – localised	28 (15.1%)
Congestive heart failure	19 (10.2%)
Myocardial infarction	17 (9.1%)
Connective tissue disease	13 (7%)
Degenerative neurological condition	9 (4.8%)
Haematological malignancy	8 (4.3%)
Solid tumour – metastatic	7 (3.8%)
Peptic ulcer disease	7 (3.8%)
Liver disease	6 (3.2%)
Peripheral vascular disease	4
eGFR at presentation; *n* (%):	
⩾60	100 (53.8%)
45–59	30 (16.1%)
30–44	28 (15.1%)
15–29	21 (11.3)
<15	7 (3.8%)
Referral source; n (%):	
Ward	165 (88.7%)
Receiving unit	17 (9.1%)
High dependency unit	4 (2.2%)
Days from admission until palliative care referral; median [IQR]	4 [2,12]
Days of palliative care involvement; median [IQR]	2 [1,4]
Number of palliative care reviews; median [IQR]	2 [1,4]
Outcome; *n* (%):	
Death	150 (80.6%)
Any other outcome	36 (19.4%)
Ongoing palliative care	3 (1.6%)
Discharged stable:	30 (16.1%)
to ward	18
home	12
Discharged for ongoing end of life care:	3 (1.6%)
home	1
to ward	1
to hospice	1
Discharged with palliative care follow up, *n* (%)	9 (4.8%)
Days from referral to death; median [IQR]	2 [1,4]
Days from COVID-19 diagnosis to death; median [IQR]	6 [4,10]

In-patient ward referrals accounted for 165 of all referrals; a further 17 were referred from short-stay admission units. Only four were referred from high-dependency units and there were no referrals from intensive care. Palliative Care were involved for a median of 2 days [IQR 1,4] and conducted a median of two reviews [IQR 1,4]. 150 (80.6%) of patients died. The median number of days between COVID-19 diagnosis by swab and death was six [IQR 4,10]. Thirty patients were discharged from Hospital Palliative Care as symptoms stabilised such that ongoing specialist input was not required. Hospital Palliative Care were directly involved in the care of 150 (37%) of the 405 in-patients who died of COVID-19 in Greater Glasgow and Clyde in this time period.

In comparison to a ‘typical’ Hospital Palliative Care caseload in 2019 (Table 2), the COVID-19 cohort were older (median age 76 vs. 73, *p* < 0.001) under Palliative Care for a shorter time (median duration 2 vs. 5 days, *p* < 0.001) and had a higher rate of death (80.6% vs 30.4%, *p* < 0.001).

**Table 2.** Comparison table of demographics and duration of involvement for COVID-19 cohort (2020) and ‘typical’ caseload cohort (2019).

	2020 – COVID-19	2019 – all referrals	Statistical test; *p* value
Total number of patients	186	437	
Age, years; median [IQR]	76 [71,84]	73 [64,81]	−4.295^a^; *p* < 0.001
Sex, Male: female (% male)	98: 88 (52.7%)	201: 236 (46%)	2.341^b^; *p* = 0.13
Days of palliative care involvement; median (range) [IQR]	2 (0-24) [1,4]	5 (0-44) [2,9]	6.256^a^; *p* < 0.001
Outcome; n (%):	(n = 186)	(n = 389)	127.992^b,c^; *p* < 0.001
Death	150 (80.6%)	118 (30.3%)	
Any other outcome	36 (19.4%)	271 (69.7%)	
Discharged home	13 (7%)	139 (35.7%)	
Discharged to ward	19 (10.2%)	91 (23.4%)	
Discharged to hospice	1 (0.5%)	25 (6.4%)	
Transfer to another hospital	0	16 (4.1%)	
Ongoing palliative care	3 (1.6%)	0	

a Mann–Whitney *U* test for independent samples.

b Pearson chi-square test.

c Comparison is between ‘death’ and ‘any other outcome’.

Dyspnoea and agitation were the most prevalent symptoms in the COVID-19 cohort (Table 3). 140 patients were prescribed CSCI for symptom control; 121 were prescribed both an opioid and midazolam. For opioids, the median daily subcutaneous morphine equivalent final dose was 15 mg [IQR 10,20] (range 5–90). For midazolam, the median final daily dose was 10 mg [IQR 10,20] (range 2.5–60). Clinical impression of efficacy was deemed effective (symptoms improved, and no further titration required) or partially effective (improvement in symptoms but further titration advised) in 78.6% and 19% of 126 cases, respectively. CSCI pumps were stopped in seven cases due to improvement in clinical condition.

**Table 3.** Symptoms, drugs used in CSCI and clinical impression of efficacy in patients with COVID-19 referred to HPCT.

Symptoms recorded (in 169 patients); *n*:	
Dyspnoea	116
Agitation	82
Pain	35
Delirium	18
Cough	15
Anxiety	12
Fever	11
Secretions	10
Nausea and vomiting	11
Fatigue	6
Drowsiness	4
Other*	5
Number of symptoms recorded per patient; median [IQR]:	2 [1–2]
5	2
4	4
3	27
2	75
1	61
Continuous subcutaneous infusion (CSCI) used for symptom control; *n* (%)	140 patients (75.3%)
Drugs given by CSCI; *n*:	
Morphine & midazolam	62
Alfentanil & midazolam	19
Morphine, midazolam & hyoscine butylbromide	10
Oxycodone & midazolam	9
Morphine, midazolam & levomepromazine	7
Alfentanil & midazolam & haloperidol	4
Alfentanil, midazoalm & hyoscine butylbromide	3
Oxycodone, midazoalm & hyoscine butylbromide	3
Oxycodone, midazoalm & hyoscine butylbromide	3
Alfentanil alone	3
Morphine alone	3
Other**	14
Drug dose in 24 hours; median, (range) [IQR]	
All opiates in sub cut morphine equivalent (*n* = 133)	15 mg (5–90) [10, 20]
Morphine (*n* = 87)	15 mg (5–90) [10, 20]
Oxycodone (*n* = 15)	10 mg (5–40 [8, 17.5]
Alfentanil (*n* = 33)	900 mg (300–4000) [500, 1000]
Midazolam (*n* = 125)	10 mg (2.5–60) [10, 20]
Haloperidol (*n* = 4)	1.75 mg (1–2)
Hyoscine butylbromide (*n* = 21)	60 mg (40 120)
Levomepromazine (*n* = 16)	15 ( 100)
Clinical impression of efficacy (126 cases); *n* (%):	
Effective	99 (78.6%)
Partially effective	24 (19%)
Not effective	3 (2.4%)
Cases where CSCI stopped due to clinical improvement; *n*	7

* sore mouth 1, constipation 1, seizure 1, anorexia 1, muscle spasm 1.

** Morphine, midazolam, levomepromazine & hyoscine butylbromide (2). Midazolam alone (2). Morphine & metoclopramide (1). Alfentanil & metoclopramide (1). Morphine & levomepromazine (1). Alfentanil, midazolam, levomepromazine & hyoscine butylbromide (1). Alfentanil, levomepromazine & hyoscine butylbromide (1). Morphine & hyoscine butylbromide (1). Alfentanil, midazolam & metoclopramide (1). Levomepromazine alone (1). Drug info not available (2).

## Discussion

### Main findings

The median age of our COVID-19 cohort was lower than previous smaller studies,^7–9^ which may reflect the high degree of comorbidity and lower life expectancy in the west of Scotland.^10^ Dyspnoea and agitation were the most common symptoms and thus an opioid and benzodiazepine were used in most cases requiring a CSCI. The doses used for dyspnoea management and sedation were low and there was a high degree of efficacy. It reassures us that commencing CSCI promptly with standard doses initially and titrating, is effective and appropriate. This is in keeping with national guidance and existing research.^11–13^ There were a small number of patients in this study who had pre-existing Palliative Care needs in addition to those related to COVID-19. This might account for some of the higher dose ranges of medication used.

For patients with COVID-19, time spent under Palliative Care was short (median 2 days compared to 5 days). This reflects the significantly higher death rate and a short dying phase associated with COVID-19.

Despite a large number of patients with COVID-19 being referred to Hospital Palliative Care the total number of patients referred was not increased compared to the previous year. This suggests that the COVID-19 caseload replaced ‘typical’ Palliative Care patients during this phase of the pandemic.

### Strengths and weaknesses/limitations of the study

Our service evaluation is limited to hospitalised patients referred to Palliative Care. Of note, there were very few patients referred from high dependency, and none from intensive care. Additional research is required into the needs of other patients, including those in the community and those dying in hospital who are not referred to Palliative Care. Data were collected retrospectively. Data on symptom burden and CSCI efficacy were limited by the quality of written contemporaneous notes. No validated scales were used to define symptom presence or intensity. As such; differentiation between symptoms, such as agitation and delirium, was based on clinician assessment. Whilst assessment of efficacy was made by a Palliative Care specialist, formal outcome measures were not used and impact on quality of life was not available.

### What this study adds

Our findings contribute to the emerging evidence base on patient demographics, clinical profiles and Palliative Care requirements in end stage COVID-19. Our study demonstrates a short dying phase in deaths from COVID-19, with a median of 6 days from diagnosis to death and a median of just 2 days from referral to Palliative Care to death.

The replacement of ‘typical’ Palliative Care patients raises the question as to the location of these patients and the implications, for patients and services, if they present at a later stage of their illness instead. It may also suggest a need for directing more services into community management of patients in a pandemic situation. This would be an interesting area for further evaluation and research.

Pandemics characteristically have peaks of infection across disparate nations at different times and can also have multiple peaks of infection and death across time.^14^ In a globalised and connected healthcare community, our findings will provide important information to other clinicians managing patients with end stage COVID-19 disease, both during the current and likely future waves of the disease.

## References

[1] World Health Organization. WHO Director-General’s opening remarks at the media briefing on COVID-19 - 3 March 2020, https://www.who.int/dg/speeches/detail/who-director-general-s-opening-remarks-at-the-media-briefing-on-covid-19—3-march-2020

[2] Docherty AB Harrison EM Green CA, et al Features of 20 133 UK patients in hospital with covid-19 using the ISARIC WHO Clinical Characterisation Protocol: prospective observational cohort study. BMJ 2020; 369: m1985. 32444460 10.1136/bmj.m1985 PMC7243036

[3] Radbruch L Knaul FM de Lima L, et al The key role of palliative care in response to the COVID-19 tsunami of suffering. Lancet 2020; 395: 1467–1469. 32333842 10.1016/S0140-6736(20)30964-8 PMC7176394

[4] Leong IY Lee AO Ng TW, et al The challenge of providing holistic care in a viral epidemic: opportunities for palliative care. Palliat Med 2004; 18: 12–18. 14982202 10.1191/0269216304pm859oa

[5] Etkind SN Bone AE Lovell N, et al The role and response of palliative care and hospice services in epidemics and pandemics: a rapid review to inform practice during the COVID-19 pandemic. J Pain Symptom Manage 2020; 60: e31–e40. 10.1016/j.jpainsymman.2020.03.029 PMC7141635 32278097

[6] Huang C Wang Y Li X, et al Clinical features of patients infected with 2019 novel coronavirus in Wuhan, China. Lancet 2020; 395: 497–506. 31986264 10.1016/S0140-6736(20)30183-5 PMC7159299

[7] Lovell N Maddocks M Etkind SN, et al Characteristics, symptom management, and outcomes of 101 patients with COVID-19 referred for hospital palliative care. J Pain Symptom Manage 2020; 60: e77–e81. 32325167 10.1016/j.jpainsymman.2020.04.015 PMC7169932

[8] Sun H Lee J Meyer BJ, et al Characteristics and palliative care needs of COVID-19 patients receiving comfort-directed care. J Am Geriatr Soc 2020; 68: 1162–1164. 32329525 10.1111/jgs.16507 PMC7264665

[9] Turner J Eliot Hodgson L Leckie T, et al A dual-center observational review of hospital-based palliative care in patients dying with COVID-19. J Pain Symptom Manage. Epub ahead of print 6 May 2020. DOI:10.1016/j.jpainsymman.2020.04.031. PMC7200379 32387139

[10] McCartney G Walsh D Whyte B, et al Has Scotland always been the ‘sick man’ of Europe? An observational study from 1855 to 2006. Eur J Public Health 2012; 22: 756–760. 22021374 10.1093/eurpub/ckr136 PMC3505444

[11] Scottish Palliative Care Guidelines. End of Life Care Guidance when a Person is Imminently Dying from COVID-19 Lung Disease. 2020, https://www.palliativecareguidelines.scot.nhs.uk/guidelines/symptom-control/end-of-life-care-guidance-when-a-person-is-imminently-dying-from-covid-19-lung-disease.aspx

[12] Association for Palliative Medicine of Great Britain and Ireland. COVID-19 and Palliative, End of Life and Bereavement Care in Secondary Care: Role of the specialty and guidance to aid care. 2020, https://www.pslhub.org/learn/coronavirus-covid19/tips/covid-19-and-palliative-end-of-life-and-bereavement-care-in-secondary-care-role-of-the-specialty-and-guidance-to-aid-care-r1963/

[13] Jackson T Hobson K Clare H Weegmann D Moloughney C McManus S. End-of-life care in COVID-19: An audit of pharmacological management in hospital inpatients. Palliat Med. Epub ahead of print 26 June 2020. DOI:10.1177/0269216320935361. 32588748

[14] Taubenberger JK Morens DM. 1918 Influenza: the mother of all pandemics. Emerg Infect Dis 2006; 12: 15–22. 16494711 10.3201/eid1201.050979 PMC3291398

